# Exploring biomarkers for treatment response in psoriatic arthritis: a focus on multi-omics technologies

**DOI:** 10.1038/s41397-026-00410-8

**Published:** 2026-05-15

**Authors:** Moshiur Rahman Khasru, Nahdia Afiifah Abdul Jalil, Nisha Nair, Anne Barton, Paul Martin, Pauline Ho, James Bluett

**Affiliations:** 1 Versus Arthritis Centre for Genetics and Genomics, Centre for Musculoskeletal Research, Manchester Academic Health Science Centre, University of Manchester, Manchester, United Kingdom; 2 Musculoskeletal Medicine and Interventional Physiatry Division, Department of Physical Medicine and Rehabilitation, Bangladesh Medical University, Dhaka, Bangladesh; 3 Department of Anatomy, Faculty of Medicine, Universiti Kebangsaan Malaysia, Kuala Lumpur, Malaysia; 4 NIHR Manchester Biomedical Research Centre, Manchester University NHS Foundation Trust, Manchester Academic Health Science Centre, Manchester, United Kingdom; 5 The Kellgren Centre for Rheumatology, Manchester Royal Infirmary, Manchester University NHS Foundation Trust, Manchester, United Kingdom

**Keywords:** Gene expression, Predictive medicine, Epigenetics

## Abstract

Psoriatic arthritis (PsA) is a complex inflammatory disease characterised by a combination of cutaneous and musculoskeletal symptoms, which complicates both diagnosis and management. Despite advances in targeted therapy, the treatment of PsA remains challenging, with up to 40% of patients experiencing inadequate response. Predictive biomarkers may serve as valuable tools to help clinicians select the most appropriate therapy at the optimal time. However, despite substantial research activity, the current evidence remains inconclusive. Moreover, biomarkers that predict treatment response may differ from those that dynamically change in response to therapy. In this narrative review we therefore examine both biomarkers with potential predictive value and those that demonstrate treatment-related changes over time. Multi-omics approaches have enhanced understanding of the molecular determinants of therapeutic response. Most genetic analyses have investigated TNF inhibitor response, whereas certain transcriptomic studies highlight genes related to apoptosis and immune-mediated pathways as key contributors to therapeutic response across different treatments and throughout the treatment course. Several non-coding RNAs and protein panels have also shown promise as indicators of treatment outcome, but metabolomic studies remain scarce. Reported molecular signatures nonetheless vary widely, likely reflecting disease heterogeneity as well as differences in treatment modalities, study design, and outcome measures, and are summarised herein. Future studies integrating multi-omics and computational frameworks are essential to validate candidate biomarkers and facilitate personalised treatment strategies in PsA.

## Introduction

Psoriatic arthritis (PsA) is a chronic inflammatory disorder associated with significant joint damage and functional impairment [1, 2]. Approximately, 10-30% of individuals with psoriasis (PsO) develop PsA, typically manifesting within a decade following the onset of skin symptoms [3]. PsA is characterised by its heterogeneity and complexity, exhibiting multiple patterns of joint involvement, including both axial and peripheral disease, as well as extra-articular manifestations such as enthesitis, dactylitis, and uveitis [2]. The Classification Criteria for Psoriatic Arthritis (CASPAR) are the most widely endorsed classification criteria for PsA, offering a high sensitivity of 99.7% and specificity of 99.1%, thereby providing a robust and reliable method to classify this complex condition [4]. There remains a lack of specific laboratory tests for the diagnosis of PsA and approximately 15% of patients with PsA remain undiagnosed. Moreover, the lack of implementation of screening questionnaires may contribute to delays in diagnosing PsA [5]. Despite therapeutic advances, predicting treatment response in PsA remains a major challenge with up to 40% of patients exhibiting an inadequate response to the treatment. The critical unmet need is for validated biomarkers to guide treatment selection, facilitating a shift from trial-and-error to a precision medicine approach for improved patient outcomes [6]. Beyond predicting treatment response, the application of omics technologies holds significant promise, enabling the generation of comprehensive molecular profiles and the recognition of pathways or biology that deepen our understanding of treatment response [7].

## Current treatment strategies and response to treatment in PsA

The overarching goal of treating patients with PsA is optimizing health-related quality of life, which ideally means achieving at least minimal disease activity, preferably remission, preventing structural damage, and maintaining a good quality of life [8]. Current European Alliance of Associations for Rheumatology (EULAR) and National Institute for Health and Care Excellence (NICE) guidelines recommend that clinicians prescribe conventional synthetic disease-modifying anti-rheumatic drugs (csDMARDs), such as methotrexate (MTX), as the first-line treatment [8, 9]. The anti-inflammatory action of low-dose methotrexate is mediated primarily via adenosine release, which suppresses pro-inflammatory cytokines. It may also modulate JAK-STAT and NF-κB signaling [10]. If the patient continues to experience high disease activity despite these therapies, switching/escalating to a biologic or targeted synthetic (b/ts) DMARD therapy is the next therapeutic option [9]. However, if this approach fails, transitioning to a treatment with a different mechanism of action is recommended [8]. Tumour necrosis factor inhibitors (TNFi) are a class of biologic therapies targeting tumour necrosis factor-alpha (TNF-α), a key mediator in the pathogenesis of PsA, driving inflammation and immune dysregulation [2]. TNFi include adalimumab, etanercept, infliximab, golimumab, and certolizumab, licensed for use in PsA. These therapies have been shown to reduce inflammation, improve symptoms, and limit structural joint damage [11]. As reported by Mitoma et al. beyond the shared neutralisation of soluble TNF-α, each anti-TNF agent possesses distinct pharmacological properties dictated by its structure [12]. Only full IgG1 monoclonal antibodies (infliximab, adalimumab, golimumab) mediate Fc-dependent effects like complement-mediated cytotoxicity (CDC) and antibody-dependent cellular cytotoxicity (ADCC), while etanercept uniquely neutralises lymphotoxin-alpha [12]. In contrast, the PEGylated Fab’ certolizumab lacks an Fc region, forfeiting cytotoxic functions but enhancing tissue penetration. Fc presence also prolongs half-life via FcRn recycling. These structural distinctions in neutralisation potency, effector functions, and pharmacokinetics underpin the agents’ divergent clinical efficacy profiles [12]. Moreover, heterogeneity in reported biomarkers and genes across studies may be explained by distinct PsA immune endotypes associated with TNFi response and by tissue-specific molecular programmes in synovium, skin, and blood [13, 14]. For instance, evidence suggests that etanercept is less effective for skin disease compared to adalimumab in PsA, although direct head-to-head trials are lacking [15]. In addition, variability in outcome definitions, timepoints, and the inclusion of small, heterogeneous cohorts may further account for inconsistent findings across studies [16].

The Interleukin (IL)-23/IL-17 axis plays a pivotal pathogenic role by integrating genetic risk with dysregulated innate and adaptive immune responses, leading to inflammation and structural damage in joints, entheses, skin and bone [17, 18]. IL-23 acts upstream to maintain and expand pathogenic IL-17–producing immune cells, while IL-17 functions as a key effector cytokine that promotes synovitis, enthesitis, keratinocyte hyperproliferation and aberrant bone remodelling [17, 18]. Interleukin (IL)-17A inhibitors (IL-17Ai; secukinumab, ixekizumab) and the dual IL-17A/F inhibitor (IL-17A/Fi; bimekizumab) provide effective control of key PsA domains including peripheral and axial arthritis, skin disease, enthesitis, and dactylitis by directly neutralising downstream IL-17 effector signalling [19, 20]. In contrast, the IL-12/23 inhibitor (IL-12/23i; ustekinumab [p40 subunit]) and IL-23 p19 subunit inhibitors (IL-23p19i; guselkumab, risankizumab) act upstream by inhibiting IL-23 signalling, thereby limiting the activation and maintenance of IL-17 producing cells and improving both musculoskeletal and skin outcomes [21–24]. However, in axial disease, IL-17 production occurs partly independently of IL-23, especially in the spine and sacroiliac joints, which may explain why IL-23 inhibitors show limited efficacy in axial involvement [25, 26].

Targeted synthetic DMARDs (tsDMARDs), comprising Janus kinase inhibitors (JAKi) and the phosphodiesterase-4 (PDE4) inhibitor apremilast, modulate immune responses through distinct intracellular mechanisms. JAK inhibitors (tofacitinib and upadacitinib) selectively inhibit JAK enzyme activity, thereby disrupting cytokine receptor–mediated signal transduction, preventing STAT phosphorylation and nuclear translocation, and ultimately attenuating pro-inflammatory gene expression [27]. Conversely, apremilast enhances intracellular cyclic adenosine monophosphate (cAMP) levels, leading to activation of protein kinase A and downstream suppression of inflammatory mediator production [28]. Both pathways offer oral, targeted approaches to controlling inflammation in PsA.

Overall, prioritisation among b/tsDMARDs should be guided by mechanism of action and dominant disease domains: IL-17Ai/IL-17A/Fi, IL-23p19i, or IL-12/23i for prominent skin disease; adalimumab or other TNFi monoclonal antibodies for uveitis; adalimumab, infliximab, certolizumab, and ustekinumab with a consideration of guselkumab for Crohn’s disease; and adalimumab, infliximab, golimumab, ustekinumab, and tofacitinib with a consideration of upadacitinib for ulcerative colitis, with avoidance of IL-17 pathway inhibition in active inflammatory bowel diseases [9]. In axial disease, IL-17Ai may represent a more effective option, whereas IL-23 inhibitors should be avoided [8, 9].

Despite the proven benefits of remission with targeted therapies, fewer than 40% of patients, attain minimal disease activity [29]. Moreover, patients who respond poorly to their first biologic are less likely to respond to subsequent therapies [30]. During periods of non-response, the disease itself may progress, leading to poorer outcomes [31, 32]. Different disease domains may become active at different times, and the variability in domain involvement and treatment response can complicate disease management [33]. Lack of response to treatment in PsA may be attributed to several factors, potentially reflecting the diverse activities of the inflammatory drivers and pathways across different tissues or domains, making a one-therapy-fits-all strategy challenging to achieve. Additionally, obesity, fibromyalgia, depression, and non-adherence are contributing factors to reduced response to treatment [34].

## Characteristics of an ideal theranostic biomarker

In an ideal world, a biomarker test would be used to accurately predict patient’s response to the treatment even before therapy begins (theranostic biomarker), enabling timely intervention to slow or halt disease progression. An ideal biomarker would guide early treatment stratification of patients, offering reliable insights into which patients could proceed directly to the next line of treatment, skipping the other lines of therapy. Consequently, clinicians would be able to prescribe the most effective treatment from the start, without delays or using a trial-and-error process. Current guidelines recommend selecting treatments based on cost, but these will not be effective in all patients [9]. Administering more expensive drugs early, if proven to be effective, would be more beneficial than waiting for non-response to occur which may incur higher overall healthcare costs [35]. A theranostic test does not need to be a single entity; it could take the form of an assay comprising multiple individual biomarkers, which, when combined, have higher predictive accuracy [36]. Finally, the test should be specific, sensitive, robust and widely accessible across healthcare centres.

## Biomarker identification through multi-omics approaches

Interpreting the complexity of PsA is greatly enhanced by the application of omics technologies, which enable unbiased exploration of multifaceted conditions. Omics technologies, encompassing genomics, transcriptomics, epigenetics, proteomics, and metabolomics, represent systematic approaches for exploring biomarkers predictive of treatment response. Through high-throughput profiling of molecular alterations, omics technologies can facilitate the characterisation of inter-individual variability in drug efficacy and response. Next-generation sequencing (NGS) provides rapid genome sequencing, enabling comprehensive identification of genetic variation. RNA sequencing (RNA-seq) and microarrays quantify gene expression, detect splice variants, and facilitate analysis of regulatory networks. Epigenetic analyses reveal DNA methylation, histone modifications, and non-coding RNA networks that regulate gene activity. Proteomics profiles protein expression and post-translational modifications, and metabolomics captures metabolic signatures. Integrating omics data enhances biomarker signature sensitivity and specificity. Bioinformatics tools are essential for analysing large datasets, requiring advanced computational methods to manage their complexity. In this review, we highlight the progress of the omics-driven therapeutic response biomarker discovery in PsA, including those that vary with treatment over time.

### Genetic biomarkers for predicting treatment response in PsA

Genetic biomarkers are ideal candidates for biomarkers as they are inherently stable, as an individual’s genetic makeup remains unchanged, allowing for pre-treatment measurement. Moreover, decreasing costs of genotyping have made testing of genetic biomarkers economically feasible [37, 38]. Therefore, identifying and validating genetic biomarkers for treatment response in PsA offers substantial potential for advancing precision medicine [38]. Although numerous genetic studies have been published in the literature, few, to date, were hypothesis-free GWAS of response in PsA (Table 1).

**Table 1:** Genetic predictors of treatment response in PsA.

Drug	Gene/Locus	Polymorphism (rs ID)	Sample Size	Population	Findings	Reference
Methotrexate (MTX)	*MTHFR*	rs1801133	163	PsA	*MTHFR* polymorphism (rs1801133) was associated with better treatment response and higher hepatotoxicity.	[40]
	*MTHFR*	rs1801131	163	PsA	*MTHFR* polymorphism (rs1801131) was associated with lower efficacy and was negatively associated with hepatotoxicity to MTX	[40]
	*DHFR*	rs1232027	119	PsA	*DHFR* was associated with better treatment response	[39]
TNFi	*TNFAIP3*	rs610604, rs6920220	20	*PsA*	*TNFAIP3* Polymorphisms were significantly associated with quality-of-life improvement at 3 and 6 months to TNFi therapy.	[81]
TNFi	*FCGR2A*	rs1801274	103	PsA	Both homozygous and heterozygous combinations of *FCGR2A* gene had better to TNFi at 6 months.	[82]
TNFi	*TNF-R1A*	rs767455	55	*PsA*	*TNF-R1A* A/A genotype associated with better response.	[83]
TNFi	*SLCO1C1*	*rs3794271*	81	*PsA*	*SLCO1C1 gene was associated with a better treatment response*	[42]
TNFi	*TNFRSF1B*	*rs1061624*	20	*PsA*	*TNFRSF1B* gene was associated with a better response.	[42]
Adalimumab	*TNF-a*	SNP + 489AA	57	PsA	The *TNF- a* + 489AA genotype associated with the positive response to adalimumab.	[84]
Etanercept	*TNF-a*	SNP + 489GG/GA	57	PsA	*TNF- a* SNP + 489GG/GA genotype associated with the positive response to Etanercept	[84]
	*FCGR2A*	*rs1801274*	55	PsA	*FCGR2A* gene had better to Etanercept at 6 months.	[42]
Infliximab	*TRAILR1 CC*	rs20575	55	PsA	*TRAILR1* C/C was associated with improved 6-month response.	[83]
	*FCGR3A*	rs396991	16	PsA	*FCGR3A* (rs396991) A/C genotype was associated with a better response to Infliximab.	[42]

#### Conventional synthetic DMARDs (csDMARDs)

csDMARDs such as MTX have been studied for genetic predictors of response in psoriatic disease. Many of the studies have focused on genes within the MTX pathway; for example, polymorphisms of *MTHFR* genes showed variable treatment responses to PsA [39, 40]. The presence of the minor allele at rs1801133 in *MTHFR* gene was correlated with higher liver toxicity to MTX in PsA (OR = 2.52 *p* = 0.04 [39]. However, heterozygosity of rs1801131 in *MTHFR* gene has been reported to be protective for raised liver enzymes (OR = 0.46, *p* value 0.040) to MTX therapy in PsA, but, given the modest sample size, the results require further validation [40]. A variant within the *DHFR* (rs1232027) gene was associated with better treatment response measured by 50% reduction in actively inflamed joint count (OR = 2.99, *p*- value = 0.02) to MTX in PsA [39].

#### Biological DMARDs (bDMARDs)

Whilst previous GWAS in patients with RA treated with TNFi have reported that the rs7195994 variant at the *FTO* locus is associated with infliximab response, particularly changes in swollen joint count (SJC28) (*p* = 9.74 × 10⁻⁹) [41], it is noteworthy that equivalent GWAS data for TNFi response in PsA remain unavailable. However, candidate gene studies have been undertaken and are summarised in Table 1.

The table shows little overlap between the genetic variants reported. This may be because, first, different studies have targeted different candidate genes; second, some studies tested association of genetic variants with TNFi as a group, whilst others have focussed on individual TNFi and, finally, sample sizes for all studies are modest compared to RA studies [41]. The best evidence for association is with variants mapping to the TNF-alpha promoter region and the *FCGR2A* gene, where more than one study reported association.

Whilst there are no studies of genetic association with treatment response to IL17A inhibitors, IL-12/23 blockers and IL-23 inhibitor in PsA, some studies have investigated association with response in patients with PsO or inflammatory bowel disease (IBD) [42]. For instance, *HLACw6* (rs12191877), *FCGR2A* (rs1801274), *and miR-146a* (rs2431697) were associated with non-response, while *PDE4A* (rs1051738) gene was associated with reduced risk for non-response to Secukinumab in PsO [43]. However, genetic variations in the protein-coding and untranslated regions of the *IL-17A* gene did not associate with response to IL-17A inhibitors in PsO patients [44].

#### Targeted synthetic DMARDs (tsDMARDs)

There is no genetic data for treatment response to JAKi and PDE4 inhibitors in PsA but a GWAS conducted on 34 Russian patients with psoriasis (PsO) identified that the *IL1β* (rs1143633), *IL4* (rs20541), *IL23R* (rs2201841), and *TNFα* (rs1800629) variants were associated with better treatment response [45].

### Therapeutic response transcriptomic biomarkers

The RNA transcriptome reflects a cell’s state, providing insights into gene function, regulation, genome flexibility, transcript changes, and therapy response [46]. Although transcript-based biomarker studies on treatment response remain limited, they have enhanced the understanding of PsA by identifying molecular signatures associated with disease suppression and remission. Variability in tissue sources, outcome measures, and transcriptomic platforms used in studies hinders the ability to integrate findings, limiting the consolidation of predictive biomarkers for treatment response. Nonetheless, several key themes have emerged, including aspects of immunopathology (interleukins, CD4 + , B, T and natural killer cells) [47, 48]; inflammation (involving TNF, TGF-β, NF-κB pathway, and chemokines) [49–51]; angiogenesis, platelet activation [52] as well as considerations related to bone metabolism [50] and apoptosis [53]. These findings not only highlight ongoing efforts to elucidate the molecular mechanisms underlying treatment responses in PsA but also shed light on the potential pathophysiology of the disease [52]. Results of transcriptomic studies reported, to date, in PsA are summarised in Table 2.

**Table 2:** Potential biomarkers for predicting therapeutic response in PsA to various treatments.

Drugs	Genes	Pathways/Biological process	Disease activity index	Durations	Methods	Sample	Reference
Adalimumab	*BNIP3, BNIP3L, BCL2L1*	Apoptosis	PASI, BSA	24 months	RT-qPCR	PBMCs	[55]
Adalimumab	*MAP2K2*	MAPK pathway	PASI, DLQI, BSA, DAS28	3 months	oligonucleotide microarrays and RT-qPCR	PBMCs	[54]
Adalimumab	*ROCK1, RhoA, LIMK2*	Apoptosis	PASI, DLQI, BSA and DAS28	24 months	oligonucleotide microarrays and RT-qPCR	PBMCs	[53]
Adalimumab/ Etanercept	*TNFR1, TNFR2*	TNF	PASI, BSA, DAS28	Every 12 weeks	RT-qPCR	Whole blood	[56]
TNFi	*FOS, CCDC50, KLRB1*	bone metabolism, inflammation	DAPSA	-	DNA microarray, RT-qPCR	Whole blood	[50]
IL-17Ai	*RAC1, ROCKs*	Rho GTPase signalling pathway	DAPSA	3 months	RNA-seq	CD4^+^ T cells	[47]
Secukinumab	*CCL4L1, CXCL10*	IL-8, toll-like receptor, apoptosis, EIF2, macrophage inhibitory factor, protein kinase A	PsARC, PASI, ACR20	Every 12 weeks	RNA-seq	Peripheral blood neutrophils,	[49]
Ustekinumab	*TGF-β1, TGF-β2, TGF-β3*	TGF-β	-	week 16, 28 and 40	RT-qPCR	PBMCs	[51]
Guselkumab	Baseline - Genes related to neutrophil, mononuclear cell and CD11+b At 24 weeks – genes associated with B and T cell, natural killer cells	Innate and adaptive immune response	ACR20, PASI75	Week 4 and 24	RNA-seq	Whole blood	[48]
DMARDs, biologic	-	Dyamic cross-talk between blood immune cells and skin fibroblasts	ACR50, DAPSA	0,1 & 6 months	RNA-seq	Whole blood and skin	[52]

*ACR50* American college of rheumatology 50% improvement, *BSA* body surface area, *DAPSA* disease activity in psoriatic arthritis score, *DAS28* disease activity score (28 joints), *DLQI* dermatology life quality index, *IL-17Ai* interleukin-17A inhibitor, *PASI* psoriasis area and severity index, *PASI75* psoriasis area and severity index 75% improvement, *PBMCs* peripheral blood mononuclear cells, *RT-qPCR* reverse transcription quantitative polymerase chain reaction, *TNF* tumor necrosis factor, *TNFi* tumor necrosis factor inhibitor.

#### Prediction of treatment response

Three studies have analysed the transcriptomic profile of PBMC samples in patients with PsA treated with adalimumab and found apoptosis-related genes (*BNIP3, ROCK1, RhoA and LIMK2*) and mitogen-activated protein kinases (*MAPK*) pathway gene expression to be correlated with treatment response [53–55]. In another study, *TNF-α, TNFR1*, and *TNFR2* were identified as potential predictive markers for response to adalimumab and etanercept [56].

At the beginning of treatment with interleukin-17A inhibitor (IL-17Ai) or TNFi, transcriptomic analysis of CD4+ cells identified over 100 differentially expressed genes (DEGs) that distinguished responders from non-responders. Following detailed network and pathway analyses, Rho GTPase signalling pathway, specifically associated with the IL-17Ai response, along with other genes (*RAC1* and *ROCKs*), suggesting that these are important components driving pathway rewiring in response to treatment [47].

#### Biomarkers of early response and non-response

Whilst several studies have primarily examined predictive biomarkers corelated with treatment outcome, Grivas et al. reported that as early as one month of initiating treatment in PsA patients, genes associated with platelet activation and MST1-mediated Hippo signalling could distinguish between responders and non-responders to DMARDs or biologic. This finding suggests the potential of these signature genes to serve as early transcriptomic biomarkers with prognostic value [52]. Conversely, poor response in PsA involved upregulated TGFβ and angiogenesis genes and increased non-classical monocytes.

A longitudinal study tracked changes in inflammatory gene expression in response to treatment, aiming to identify predictive biomarkers and explore the evolving molecular profile of PsA patients. Transforming Growth Factor-β (TGF-β) plays a crucial role in psoriatic disease pathogenesis by promoting T helper 17 (Th17) cell release, central to PsA inflammation [57]. A 40-week study of 14 PsA patients assessed TGF-β expression changes following ustekinumab therapy [51]. TGF-β1 and TGF-β2 increased during the first 28 weeks, with significant changes in TGF-β1. TGF-β3 decreased until week 16 before rising. Monitoring these changes may optimise treatment strategies.

#### Biomarkers enhancing the understanding of immunopathology of treatment response

Certain studies have characterised the immunopathological changes occurring during treatment, providing mechanistic insights into the immune processes that underpin therapeutic efficacy. A consistent observation across studies is the presence of dysfunctional immune cell profiles, highlighting the role of immune dysregulation in modulating treatment response. A transcriptomic profiling study comparing PsA patients in remission (methotrexate-resistant and receiving TNFi treatment) with healthy individuals revealed broad similarities in gene expression between the two groups [50]. The study found that out of 125 identified genes, 25 were coding genes actively participating in the immune response. Notably, *FOS* (fold change = −2.0, *p* = 0.005), a gene associated with inflammation and osteoclastogenesis, was downregulated, while *CCDC50* (fold change = +1.5, *p* = 0.005), which plays a role in inhibiting osteoclastogenesis, was upregulated in patients who responded well to the therapy. The regulation of these genes may be important for maintaining a balance between ongoing disease activity and remission, highlighting their potential role in therapeutic response and disease modulation [50].

Guselkumab treatment has been shown to influence the expression of immune cell-related genes in PsA [42]. Initially, prior to drug administration, 355 genes were upregulated; of these, 82% (293 genes) were downregulated after 24 weeks of treatment, while 77% of the 314 genes that were initially downregulated (242 genes) became upregulated by week 24. Notably, among the top upregulated genes at week 24 were HLA-DOA, TNFRSF13C, and CD1C, whereas APMAP, KREMEN1, and GYG1 were downregulated. Subsequent gene set enrichment analysis revealed that the upregulated PsA-associated genes at week 24 were related to T cells, B cells, and natural killer cells. This study highlights the significant DEGs associated with guselkumab therapy, which are linked to a dysfunctional immune profile, and underscores the importance of the immunopathological mechanisms underlying treatment response in PsA [48].

### Epigenetic biomarkers

Epigenetic mechanisms, which regulate gene expression without altering the underlying DNA sequence, play a key role in mediating the interaction between genetic and environmental factors in psoriatic disease and in maintaining heritable patterns of gene transcription [58].

#### DNA Methylation

DNA methylation has been investigated as a potential biomarker for disease diagnosis and treatment response; however, the evidence to date remains limited. In one study of PsA patients, treatment with MTX increased global DNA methylation, suggesting that hypomethylation is a characteristic of inflammation [59].

#### Non-coding RNAs

More than 95% of the eukaryotic genomic is transcribed into non-coding RNAs (ncRNAs), which play a crucial role in regulating gene expression. Among these, microRNAs (miRNAs), small single stranded, non-coding RNA molecule, regulates diverse biological activities by interacting with mRNA [60]. Inflammation processes can dysregulate miRNAs expression by disrupting the transcription of precursor transcripts or compromising the stability of mature miRNAs. Moreover, increasing research has focused on the regulatory roles of miRNA in the immunological disorders and their potential therapeutic applications [61]. Given their involvement in the pathogenesis of autoimmune diseases, miRNAs are now being increasingly investigated as potential biomarkers for predicting therapeutic response [62]. Long non-coding RNAs (lncRNAs) are transcripts of 200 nucleotides that do not possess any protein-coding function. A growing body of evidence reveals that aberrant lncRNA expression contributes immune system dysregulation and inflammatory process in various autoimmune diseases [63]. Table 3 summarises the findings of ncRNAs as predictive biomarker of therapeutic response in PsA.

**Table 3:** Potential ncRNA biomarkers for predicting therapeutic response.

Drugs	ncRNA	Pathways/Biological process	Disease activity index	Duration	Method	Sample	Reference
DMARD	miR-221-3p, miR-130a-3p, miR-146a-5p, miR-151-5p, miR-26a-5p, and miR-21-5p	Immunology	EULAR response, VAS-100	3, 6, 9 months	miRNA assay	Blood serum	[64]
methotrexate, etanercept, low dose steroid& cyclosporin	miR-21-5p	inflammatory and immune system	DAPSA, DAS28-ESR	12 weeks	MicroRNA microarray	Whole blood	[65]
Methotrexate	miR-21-5p	IL-17/IL-23 axis and CXCL10 pathways	Clinical disease activity	24weeks	Droplet digital PCR	Serum and whole blood	[66, 85]
Methotrexate	miR-127-3p, miR-155-5p, miR-140-3p, miR-432-5p, miR-382-5p, miR-532-5p, miR-139-3p, miR-379-5p	MAPK and WNT signaling	DAPSA	6 months	next-generation sequencing	Serum	[67]
TNFi or IL-17i	MEG3	cell cycle regulation, proliferation process and apoptosis	DAPSA	6 and 12months	lncRNA PCR array	PBMCs	[68]

*DAPSA* disease activity in psoriatic arthritis score, *DAS28-ESR* disease activity score in 28 joints – erythrocyte sedimentation rate, *DMARD* disease-modifying antirheumatic drug, *EULAR* European league against rheumatism, *IL-17Ai* interleukin-17A inhibitor, *lncRNA PCR* long non coding RNA polymerase chain reaction, *PBMCs* peripheral blood mononuclear cells, *TNFi* tumor necrosis factor inhibitor, *VAS-100* visual analogue scale (0–100 mm).

##### miRNA

In early PsA, six miRNAs (miR-221-3p, miR-130a-3p, miR-146a-5p, miR-151-5p, miR-26a-5p, and miR-21-5p) were reported to be differentially expressed, with higher levels in patients exhibiting greater disease activity [59]. Baseline levels of miR-130a-3p, miR-221-3p, miR-146a-5p, miR-151a-5p, and miR-26a-5p predicted treatment response, independent of medication type (biologics or DMARDs) or disease duration. Higher baseline miRNA expression correlated with better therapeutic outcomes. ROC analysis showed miR-221-3p, miR-130a-3p, and miR-146a-3p effectively distinguished responders from non-responders (AUC > 0.7) [64]. In another study, miR-21-5p was significantly downregulated in PsA patients after 12 weeks of therapy and correlated with improved DAPSA scores [65].

The route of MTX administration has been reported to influence miR-21-5p expression, with subcutaneous MTX downregulating miR-21-5p and correlating with clinical improvement [66]. Lower pre-treatment levels of miR-127-3p were associated with joint response, with strong predictive accuracy (AUC = 0.9). For skin response, seven miRNAs (miR-155-5p, miR-140-3p, miR-432-5p, miR-382-5p, miR-532-5p, miR-139-3p, miR-379-5p) were identified, with miR-155-5p demonstrating an AUC of 0.76. miR-127-3p targets key PsA pathways, including MAPK and WNT signalling, highlighting its potential as a biomarker of joint response [67].

##### Long non-coding RNAs (lncRNAs)

lncRNAs may also play a role in PsA treatment response. A 12-month longitudinal study involving 48 PsA patients receiving TNFi or IL-17i identified differentially expressed lncRNAs associated with treatment response. PCR screening revealed differential gene expression of four lncRNAs in TNFi-treated and three in IL-17i-treated patients. Validation confirmed significantly reduced MEG3 expression in non-responders across both treatment groups [68].

### Proteomic biomarkers

Protein biomarkers predictive of response to TNFi and IL17Ai have been reported across several studies and are summarised in Table 4. Changes in serum MMP-3 levels before and after TNFi therapy have been reported to predict treatment response in PsA [69, 70]. Similarly, increases in cartilage-derived proteins such as oligomeric matrix protein (COMP) and melanoma inhibitory activity (MIA) were found to be correlated with response [69, 70]. Baseline MMP-3 were also found to demonstrate strong predictive value for biologic treatment response, together with CXCL10, S100A8, and ACP5 [71]. In another study employing gel-electrophoresis, baseline synovial expression of albumin, collagen alpha 3, and annexins A1 and A2 consistently distinguished responders from non-responders to both adalimumab and etanercept [72]. Separately, mass spectrometry–based analysis evaluated 107 candidate proteins from synovial tissue, resulting in the development of an MRM-MS assay comprising 57 proteins predictive of therapeutic response (AUROC = 0.76). Among these, S100A8 emerged as the most predictive protein, highlighting its potential key role in inflammatory and immune responses [73]. The GO–REVEAL biomarker substudy reported two protein panels predictive of response to golimumab, based on the ACR20 response criterion and the Disease Activity Score (DAS28). Both panels included adiponectin and factor VII [74]. Secukinumab response in PsA was associated with beta-defensin 2, identified as a predictive biomarker through a large-scale SomaScan-based proteomic analysis across five phase III trials. The association was further validated in the FUTURE 2 and EXCEED studies and confirmed by ELISA [75].

**Table 4:** Potential proteins biomarkers predictive of therapeutic response.

Drugs	Proteins	Disease activity index	Timepoints	Method	Sample	Reference
Adalimumab	MMP3, MIA	DAS28	baseline, 4 and 12 weeks	ELISA	serum	[70]
Adalimumab, etanercept, golimumab, infliximab & csDMARDs	MMP3, COMP	tender joint count, swollen joint, PASI	baseline, 3 months	ELISA	serum	[69]
Golimumab	Panel for ACR 20- adiponectin, factor VII, PAP, pyridinoline, Panel for DAS28- adiponectin, factor VII, leption, IgA, SGOT	DAS28-CRP, ACR20, PASI75	baseline, week 4 and week 14	ELISA LUMINEX	serum	[74]
Adalimumab, abatacept	Panel of 57 proteins, including S100-A8, S100-A10, annexin A1 and A2, α-1 acid glycoprotein, haptoglobin, Ig κ chain C fibrinogen-α and -γ, vitronectin and collagen α-2	DAS28-CRP	baseline, 4 weeks, followed up to 12 weeks	Mass spectrometry, MRM-MS assay	synovium	[73]
Etanercept, adalimumab	albumin, collagen alpha 3 and annexins A1 and A2	DAS28, ACR, EULAR	baseline, 4 and 12 weeks	gel electrophoresis-based proteomics, MRM protein assay	synovium	[72]
Secukinumab	beta-defensin 2	ACR20	baseline, weeks 1, 2 and 3 and then every 4 weeks from week 4 up to 24 weeks	SomaScan Platform, ELISA	serum	[75]
TNFi, IL-17i, methotrexate	MMP3, S100A8, ACP5, and CXCL10	PASI, DAPSA	baseline, 3-6 months	Multiplex protein quantification assay	serum	[71]

*ACR* American college of rheumatology, *ACR20* American college of rheumatology 20% improvement, *csDMARDs* conventional synthetic disease-modifying antirheumatic drugs, *DAPSA* disease activity in psoriatic arthritis score, *DAS28* disease activity score (28 joints, *DAS28-CRP* disease activity score based on the evaluation of 28 joints using C-reactive protein, *ELISA* enzyme-linked immunosorbent assay, *EULAR* European league against rheumatism, *MRM* multiple reaction monitoring protein assay, *MRM-MS assay* multiple reaction monitoring – mass spectrometry assay, *PASI75* psoriasis area and severity index 75% improvement.

### Metabolomic biomarkers

Metabolomics provides detailed insights into metabolic phenotypes, offering the potential to advance precision medicine through enhanced diagnosis, therapeutic monitoring, and biomarker discovery;however, few studies, to date, have explored the association between the metabolome and treatment response [76]. A landmark study employing nuclear magnetic resonance spectroscopy of urine samples from patients with RA and PsA demonstrated that baseline urinary metabolomic signatures—including histamine, glutamine, xanthurenic acid, and ethanolamine—distinguished responders from non-responders to TNFi therapy in RA, while both diseases exhibited significant metabolic alterations between baseline and 12 weeks of treatment [77].

## Limitations

Researchers have made extensive efforts to identify validated and robust omics-based therapeutic biomarkers for PsA. However, the findings discussed here remain in their infancy. Many of the studies reported have been performed on a small scale and are statistically underpowered, with the added challenge of high variability in disease activity, different domains of disease and outcome measure indices. The limited scale of existing studies restricts their generalisability to larger populations. Given the relatively low prevalence of PsA, international collaborations are underway to enhance the statistical power and robustness of research efforts on a global scale, with large consortia such as the Health Initiatives in Psoriasis and Psoriatic Arthritis Consortium European States (HIPPOCRATES) and the Accelerating Medicines Partnership Autoimmune and Immune-Mediated Diseases (AMP AIM) strategically working to translate these advancements into actionable clinical practice [78]. Some studies have primarily focused on known genes or omic candidates, potentially overlooking significant, yet unidentified, associations that may contribute to therapeutic responses. Despite reports of significant gene expression differences, corresponding protein levels were not measured. Furthermore, the variability in targeted therapy highlights the need for diverse studies to identify specific biomarkers for each treatment modality. Factors such as receiving concurrent treatments, adherence to prescribed therapies, and underlying metabolic conditions also serve as potential confounders that need to be accounted for in these investigations. Despite these challenges, the findings from available studies provide valuable insights and serve as a foundation for guiding future research in this field.

## Future perspectives

Future research should focus on the standardisation of multi-omic data integration and the development of robust computational frameworks to enhance predictive accuracy. Large-scale GWAS should be further expanded to identify novel genetic variants associated with drug response. Integrating transcriptomics and proteomics through the combination of RNA sequencing and proteomic profiling has the potential to elucidate pathways and gene expression signatures relevant to treatment efficacy. Moreover, the study of epigenetic modifications, such as DNA methylation and histone acetylation, may offer valuable insights into how genetic predispositions are modulated by environmental factors and therapeutic interventions. Translating these findings into clinical practice will require validation in diverse cohorts and the implementation of cost-effective diagnostic tools to facilitate routine use.

Consolidating and analysing multi-omic datasets using machine learning (ML) may offer a faster, more scalable approach to identifying disease biomarkers. ML’s ability to process high-dimensional, heterogeneous datasets enables the identification of disease biomarkers, patient stratification, and personalised treatment recommendations. By uncovering patterns and relationships that traditional methods might overlook, ML accelerates advancements in precision medicine, ultimately improving clinical outcomes and optimising care for PsA patients [79]. Additionally, longitudinal studies tracking gene expression changes during disease progression are particularly valuable, offering insights and opportunities to intervene with timely treatments. Specific disease domains should be taken into consideration in future research, with a particular emphasis on understanding multi-omic drug responses within the various domains of PsA. This approach, rather than studying responses based on the non-specific presentation of the disease, would further enhance the implementation of personalised treatment strategies for PsA.

Finally, future treatment-related research should not only prioritise therapeutic efficacy but also address treatment safety, tolerability and potential side effects such as liver injury. The ultimate goal is to identify and implement therapies that are both effective and safe, ensuring the right treatment is delivered at the right time. Achieving this balance will lead to improved patient satisfaction and enhanced quality of life.

## Conclusion

The discovery of therapeutic biomarkers in PsA requires multinational collaboration to overcome challenges posed by limited participants and low disease prevalence. A biomarker panel may offer greater clinical utility due to PsA’s complexity and the availability of multiple treatment options, as has been proposed in cancer [80]. Efforts to advance this field are already underway, with large consortia actively striving to achieve significant breakthroughs in biomarker identification and implementation.

## Data Availability

The data that support the findings of this study are publicly available in the referenced material.
